# Movements of Mycoplasma mobile Gliding Machinery Detected by High-Speed Atomic Force Microscopy

**DOI:** 10.1128/mBio.00040-21

**Published:** 2021-05-28

**Authors:** Kohei Kobayashi, Noriyuki Kodera, Taishi Kasai, Yuhei O. Tahara, Takuma Toyonaga, Masaki Mizutani, Ikuko Fujiwara, Toshio Ando, Makoto Miyata

**Affiliations:** a Graduate School of Science, Osaka City University, Sumiyoshi-ku, Osaka, Japan; b Nano Life Science Institute (WPI-NanoLSI), Kanazawa University, Kanazawa, Ishikawa, Japan; c The OCU Advanced Research Institute for Natural Science and Technology (OCARINA), Osaka City University, Sumiyoshi-ku, Osaka, Japan; d Department of Bioengineering, Nagaoka University of Technology, Nagaoka, Niigata, Japan; University of Geneva

**Keywords:** AFM, probing, pathogenic bacteria, ATPase, class *Mollicutes*

## Abstract

Mycoplasma mobile, a parasitic bacterium, glides on solid surfaces, such as animal cells and glass, by a special mechanism. This process is driven by the force generated through ATP hydrolysis on an internal structure. However, the spatial and temporal behaviors of the internal structures in living cells are unclear. In this study, we detected the movements of the internal structure by scanning cells immobilized on a glass substrate using high-speed atomic force microscopy (HS-AFM). By scanning the surface of a cell, we succeeded in visualizing particles, 2 nm in height and aligned mostly along the cell axis with a pitch of 31.5 nm, consistent with previously reported features based on electron microscopy. Movements of individual particles were then analyzed by HS-AFM. In the presence of sodium azide, the average speed of particle movements was reduced, suggesting that movement is linked to ATP hydrolysis. Partial inhibition of the reaction by sodium azide enabled us to analyze particle behavior in detail, showing that the particles move 9 nm right, relative to the gliding direction, and 2 nm into the cell interior in 330 ms and then return to their original position, based on ATP hydrolysis.

## INTRODUCTION

Many bacteria translocate to nutrient-rich places and escape from repellent substances by manipulating external appendages, such as flagella and pili (1, 2). However, class *Mollicutes*, a small group of bacteria, have as many as three of their own motility mechanisms. Class *Mollicutes* evolved from phylum *Firmicutes* by losing peptidoglycan synthesis and flagella swimming to evade host innate immunity in their parasitic life (1). They have a single-layered cell membrane featured by a high content of sterols (25 to 30% of the weight of total membrane lipids) and lipoproteins as peripheral structures (3–7). Among *Mollicutes*, the gliding motility of Mycoplasma mobile, the subject of this study, is suggested to have evolved from a combination of ATP synthase and cell adhesion (1, 5, 8–12).

**FIG 1:**
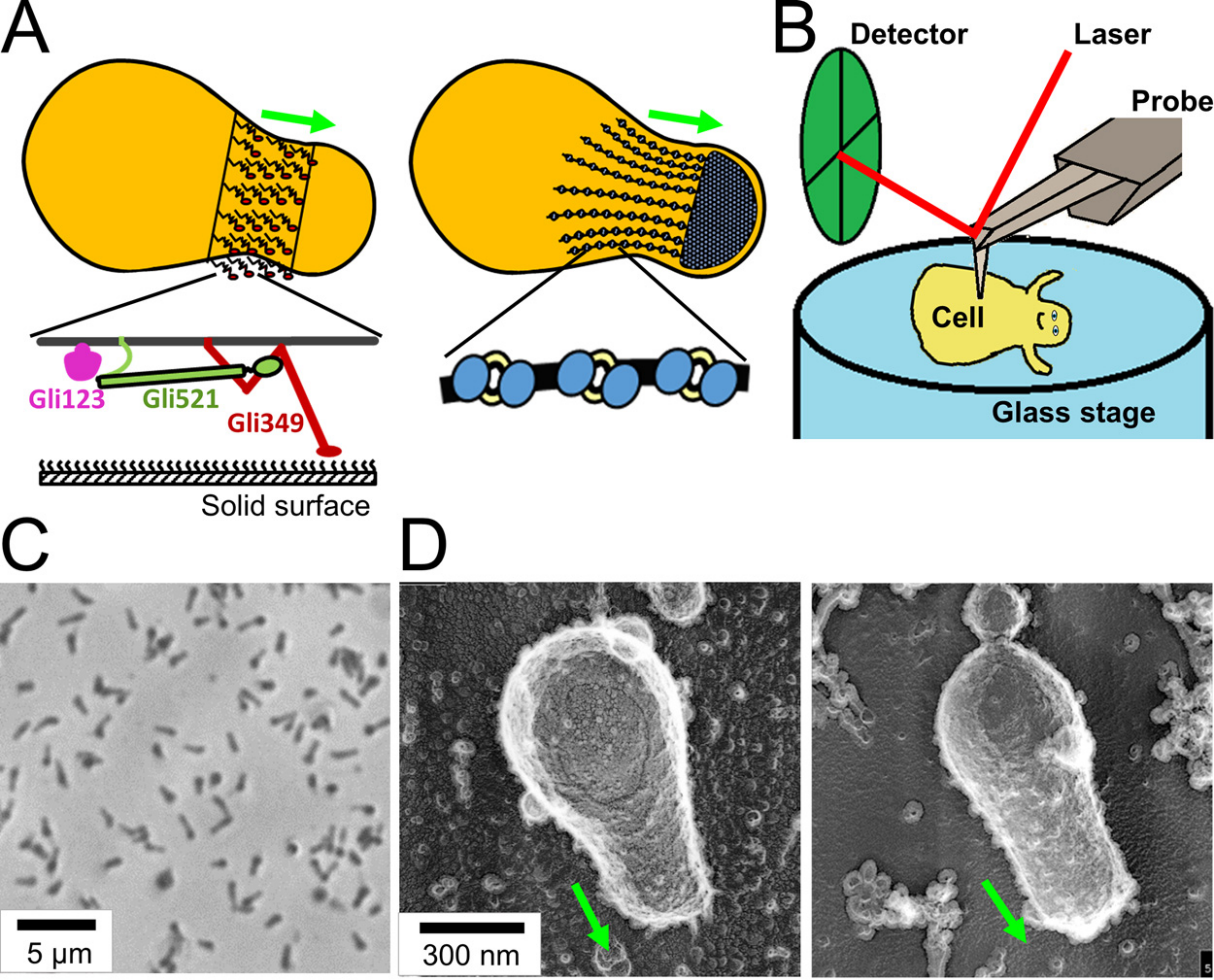
Experimental design and conditions for HS-AFM observation. (A) Schematic illustrations of *M. mobile* gliding machinery. The gliding machinery formed as a protrusion can be divided into surface (left) and internal (right) structures. The surface structure is composed of about 450 units, including three large proteins—Gli123 (purple), Gli521 (green), and Gli349 (red)—as shown at the bottom. Gli349 repeatedly catches sialylated oligosaccharides fixed on the solid surface and pulls the cell forward. The internal structure can be divided into a large mass at the cell front, “bell” and a chain structure. The chain structure is composed of particles that have been suggested to evolve from F-type ATPase/synthase. (B) Schematic illustration of an *M. mobile* cell being scanned by high-speed atomic force microscopy (HS-AFM). The surface of an immobilized cell on glass stage (blue) is scanned by an AFM cantilever probe (gray), and the cantilever movement is monitored by a detector (green). (C) Phase-contrast image of *M. mobile* cells on a coverslip. Living cells were immobilized onto a coverslip using poly-l-lysine and glutaraldehyde. (D) Quick-freeze, deep-etch electron microscopic (EM) image of *M. mobile* cells on a coverslip. The cell was immobilized on the coverslip by poly-l-lysine and glutaraldehyde (left) and allowed to glide on the coverslip coated with sialylated oligosaccharides (right). The cell axis and front are indicated by a green arrow in panels A and D.

*M. mobile*, isolated from a freshwater fish, is a flask-shaped bacterium with a length of 0.8 μm (Fig. 1A). *M. mobile* glides in the direction of its tapered end on solid surfaces, such as animal cells, glass, and plastics. Its gliding speed is 2.5 to 4 μm/s, which is 3 to 5 times its own cell length (10, 13). The gliding machinery is divided into surface and internal structures, both of which are composed of 450 units (Fig. 1A) (5, 8, 10, 14). The internal structure is characterized by multiple chains. An *M. mobile* cell has approximately 28 chains around the base of the protrusion (Fig. 1A). Each chain consists of uniformly sized particles, which are 13 nm in width and 21 nm in length (5). Interestingly, the amino acid sequence of component proteins suggests that this chain structure has evolved from ATP synthase (5, 8, 10, 12, 15). Recently, the isolated internal structure was shown to hydrolyze ATP through conformational changes, suggesting that the internal structure functions as a motor and generates the force for gliding (5, 10). The surface structure is composed of three large proteins, Gli349, Gli521, and Gli123. Gli349 has a binding site for sialylated oligosaccharide at its tip and plays the role of a “leg” in gliding (9, 16–20). Gli521 and Gli123 have been proposed to act as a “crank” that transmits force (21–24) and as a “mount” to correctly localize the surface proteins (19). A working model for the gliding mechanism has been suggested as follows (5, 10, 13, 25): the force for gliding generated based on ATP-derived energy by the special motor is transmitted across the membrane to the surface structure, including the leg structure. Then, the foot (the tip structure of the leg) repeatedly catches, pulls, and releases the sialylated oligosaccharides (9, 16), the major structures on host animal surfaces (26–28), resulting in cell migration (21, 29–32). This explains the gliding mechanism at the bacterial surface; however, the spatial and temporal behaviors and movements of internal motors in living cells have not been examined.

Atomic force microscopy (AFM) (33) is a powerful method to image the surface structures and to study the mechanical properties of a biological sample at the submolecular level (34). In this method, a sample placed on a substrate is scanned with a nanometer-scale probe under dry and wet conditions. The usefulness of this method has been demonstrated also in the field of microbiology (35, 36). In high-speed AFM (HS-AFM), the scanning speed of AFM has been dramatically improved to ∼20 frames per second (fps) while maintaining minimal invasiveness (37). Then, the dynamic behaviors of biomolecules and cells can be captured in aqueous solution (37), and their functional mechanisms have been elucidated (37–40). Notably, HS-AFM has been applied to understand the structures on the cell wall (41) or below the cell membrane (42).

In this study, we succeeded in visualizing the internal structure of *M. mobile* gliding machinery by scanning the surface of cells immobilized on a glass substrate using HS-AFM. The particle structure, a component of the internal structure, showed movements mainly in the right and inward directions relative to the gliding direction of an *M. mobile* cell.

## RESULTS

### Immobilization of living cells on the glass surface.

We attempted to visualize the gliding machinery by scanning the upper side of living cells immobilized on the substrate surface (Fig. 1B), since the gliding machinery is arranged around the base of the protruded region (Fig. 1A). Cell suspension in a buffer was placed on a glass substrate reactivated for amino groups and kept for 10 min at 25 to 28°C. Phase-contrast microscopy showed that the cells adhered to the glass substrate at a density of 1 cell per approximately 6 μm^2^ (Fig. 1C). When the buffer was replaced by growth medium containing sialylated oligosaccharides (scaffolds for gliding), half of the cells recovered to glide, suggesting that the cells were alive on the glass. Serum included in the medium contained sialylated oligosaccharides conjugated to fetuin, a serum protein. Fetuin was likely adsorbed onto the glass and worked as a scaffold for mycoplasma gliding (26–28, 43).

To observe the shape of immobilized cells, we adopted quick-freeze, deep-etch electron microscopy that visualizes cells under aqueous conditions with nanometer spatial resolution (44, 45). The morphology of immobilized cells (Fig. 1D, left) was not significantly different from that of the gliding cell visualized without any chemical fixation (Fig. 1D right).

### Visualization of immobilized cells by HS-AFM.

**FIG 2:**
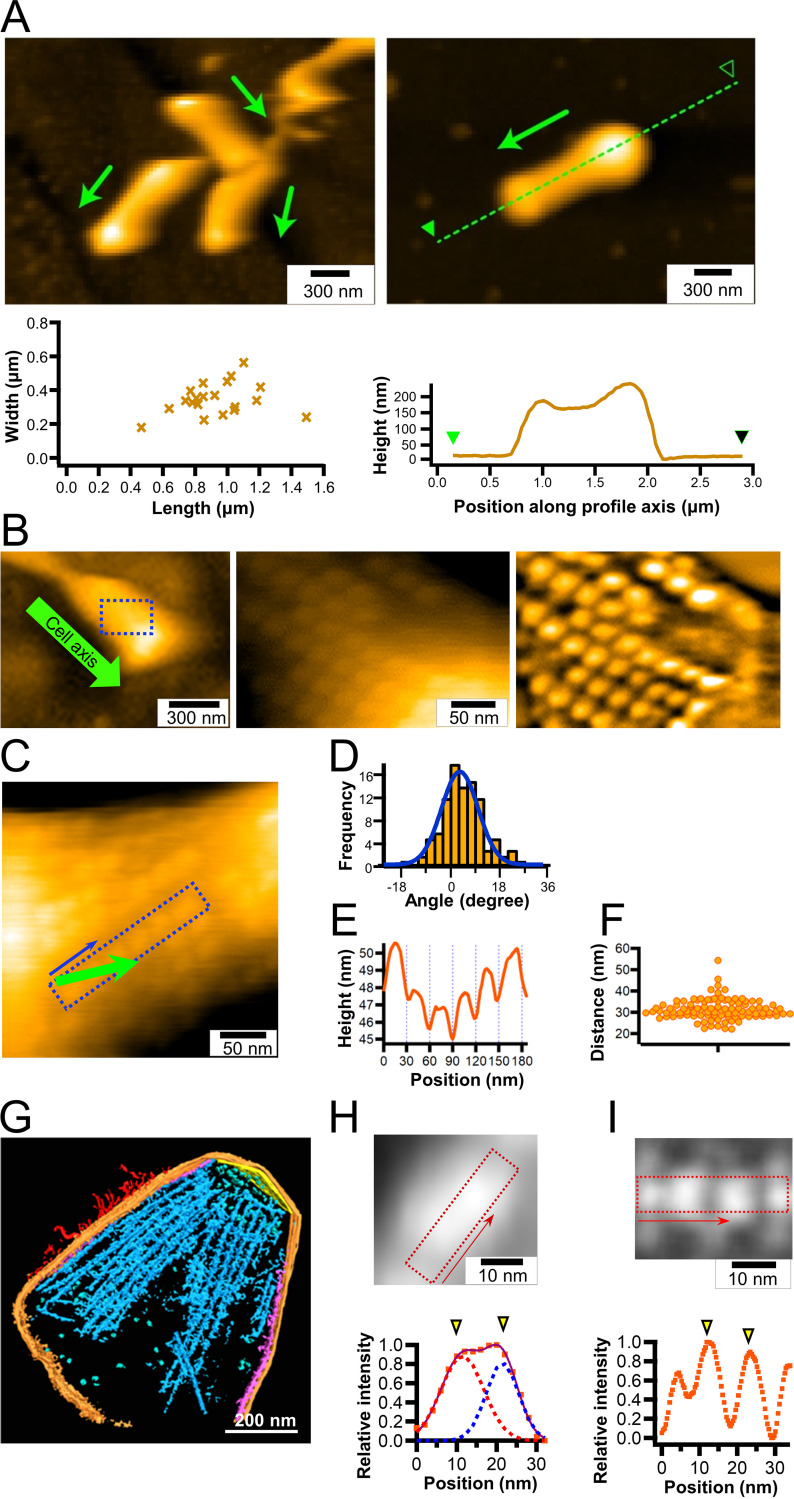
Chain imaging by HS-AFM. (A) (Left) Cluster of cells immobilized on a glass surface (upper) and distribution of cell dimensions (*n* = 20) (lower). (Right) The height profile along the broken line (upper) is plotted along the green arrow (lower). The cell axis and front are shown by an arrow. (B) Detailed structure of a cell. (Left) Whole-cell image. The cell axis and front are indicated by a green arrow. (Middle) Magnified image of the boxed area of the left panel. (Right) The middle panel image was processed with a bandpass filter. (C to F) Image analyses of particles. (C) Cell image featuring a representative chain structure. The cell axis and front are indicated by a green arrow. (D) Distribution of chain angle relative to the cell axis fitted by a Gaussian curve (*n* = 99 chains from 20 cells). (E) Image profile of the boxed area along the direction of blue arrow in panel C. (F) Scatter dot plot for distances between peak positions of the chain profile. The average was 31.5 ± 4.9 nm (*n* = 98). (G) Three-dimensional rendered image for a 146-nm-thick slice of permeabilized cell reconstructed by electron cryotomography, modified from a previous study (5). The surface filamentous structures, cell membrane, undercoating at the front and side membranes, and internal chain are colored red, orange, yellow, and purple, respectively. (H) Averaged image of 19 particle structures from HS-AFM (upper) and image profile of boxed area (lower). The profile (orange squares) was fitted by the sum (purple solid line) of two Gaussian curves (red and blue). Yellow triangles show peaks of the Gaussian curves. (I) Averaged images of chain structure (blue part in panel G) from electron cryotomography (upper) (5) and image profile of the boxed area along the chain axis (lower). Yellow triangles show peaks of Gaussian curves. In all HS-AFM imaging, the surface was scanned left to right for line and lower to upper for image.

Next, the cells immobilized on the glass surface were scanned by HS-AFM (Movie S1 and Fig. 2A). A typical *M. mobile* cell with a flask shape was found at a density of a single cell per approximately 100 μm^2^. As can be seen by comparing cell appearance in optical and electron microscopy, the cell images obtained here suggest that cells are characterized by rigidity in the front region (Fig. 1C and D), consistent with previous observations showing an internal rigid “bell” structure (5, 12). The average size of a cell was 0.93 ± 0.33 μm in length and 0.33 ± 0.08 μm in width (*n* = 20, Fig. 2A). We also measured the height along the long axis of the cell. Two peaks were found; one was near the front end, and the other was near the tail end of the cell, consistent with previously reported characteristics of *M. mobile* cells (49, 51).

10.1128/mBio.00040-21.3 MOVIE S1 HS-AFM movie searching for *M. mobile* cells. An *M. mobile* cell immobilized to the substrate surface was searched by recording at 1 fps. The video was played at a speed of 2×. The scanning field was 3 by 3 μm^2^ with 100 by 100 pixels. A cell appeared around 6 s and moved around the center of the field at 13 s. The cell front is directed to the lower right. Download Movie S1, AVI file, 0.2 MB. Copyright © 2021 Kobayashi et al. 2021 Kobayashi et al. https://creativecommons.org/licenses/by/4.0/ This content is distributed under the terms of the Creative Commons Attribution 4.0 International license.

The Young’s modulus of the *M. mobile* cells was roughly estimated to be ∼20 kPa based on the Hertz model of the spherical tip (46), by assuming that the Poisson’s ratio of the cell and the nominal radius of the tip are 0.5 and ∼5 nm, respectively. This value is comparable to that of live animal cells (10 to 100 kPa) (47), whose architecture of the cell membrane is a single lipid bilayer like *M. mobile* cells, but much smaller than that of live Escherichia coli cells (∼8 MPa) (48), which have the outer membrane and the peptidoglycan layer.

To visualize the gliding machinery, the cell surface was scanned by HS-AFM at a scanning rate of 300 ms per frame in an area of 300 nm^2^. Interestingly, we found particle structures aligned mostly along the cell axis at the front side of the cells (Fig. 2B). The particle structures appeared when the average tapping force exceeded ∼40 pN (piconewtons) (see Materials and Methods). They were aligned at an angle of approximately 4.6° relative to the cell axis (Fig. 2C and D, *n* = 99 chains from 20 cells). The particle height was approximately 2 nm (Fig. 2E), and the pitches were distributed as 31.5 ± 4.9 nm (Fig. 2F, *n* = 98), in good agreement with a previous number, 31 nm, measured by electron cryotomography (Fig. 2G) (5). To measure the dimensions of the particles in detail, we collected 19 particle images and averaged them (Fig. 2H). The averaged image showed an elliptical structure, 27.2 nm long and 14.2 nm wide, with two height peaks. The distance between the two peaks of a particle was 10.0 nm. These features were consistent with the results from electron cryotomography (Fig. 2I) (5), showing that the particle structure observed in HS-AFM is identical to the internal structure observed by electron cryotomography.

### The internal structure of *M. mobile* is detected by HS-AFM from the surface.

An *M. mobile* cell has three huge proteins, Gli521, Gli349, and Gli123, on its surface (Fig. 1A, left). To confirm that the particle structures visualized with HS-AFM are not the surface structures, the cell surface was treated with proteinase K, a serine protease with broad specificity, and scanned by HS-AFM. First, we confirmed that *M. mobile* cells gliding on the glass surface were stopped 1 min after the addition of 0.2 mg/ml proteinase K (Fig. S1A), suggesting that the surface proteins involved in the gliding machinery are sensitive to proteinase K. Then, we observed the cell surface by HS-AFM after the immobilized cells were treated with proteinase K for 20 min. The particle structures were observed on the surface of the cell even after proteinase K treatment. The particle pitches of cells with and without proteinase K treatment were 31.2 ± 3.2 (*n* = 31) and 28.9 ± 3.6 nm (*n* = 33), respectively (Fig. S1B), showing a significant difference between them (*P = *0.00651 by Student’s *t* test). Based on these observations, we concluded that the particle structure detected by HS-AFM was inside the structure but influenced by the surface treatment with proteinase K, consistent with a previous observation (12).

10.1128/mBio.00040-21.1 FIG S1 Protease treatment and unroofing confirm that particles are intracellular structures. (A) Rainbow traces of gliding cell for 5 s starting 20 s before and 60 s after the addition of 0.2 mg/ml proteinase K marked from red to purple over time. (B) Processed HS-AFM image of particle structures on a cell surface without (left) and with (middle) proteinase K treatment and scatter dot plot of distances between particles along each chain axis (right). Particle distances on cells with and without proteinase K treatment were 30.1 ± 6.1 nm (*n* = 35) and 31.2 ± 3.2 nm (*n* = 31), respectively (Student’s *t* test, *P = *0.328). (C) Time course images of HS-AFM scanning of cell membrane removal. The cell membrane started to be broken at 2 s, and the internal structure was completely exposed at 23 s after the cell was focused (*t* = 0) (Movie S2). Scanning area, 500 by 500 nm^2^ with 150 by 150 pixels; frame rate, 1,000 ms per frame. (D) Magnified HS-AFM image of particle structures before (left) and after (middle) cell membrane removal and scatter dot plot of distances between particles along each chain axis (right). The color gauge on the right side of each figure shows the scale of the relative height (the height is presented by adjusting the lowest point in panel D until it becomes 0). The averaged height of the observation surface after removal of the cell membrane was 239 nm lower than before removal (257 and 18 nm for before and after, respectively). The distances between neighboring particles before and after cell membrane removal were 30.3 ± 4.1 (*n* = 36) and 31.8 ± 7.3 nm (*n* = 40), respectively (*P = *0.277). The cell axis and front are indicated by a green arrow in panels B to D. Download FIG S1, PDF file, 0.3 MB. Copyright © 2021 Kobayashi et al. 2021 Kobayashi et al. https://creativecommons.org/licenses/by/4.0/ This content is distributed under the terms of the Creative Commons Attribution 4.0 International license.

During the observation of intact cells immobilized on glass surfaces, we observed the removal of the cell membrane by chance, resulting in the exposure of the inside structure. The exposed inside structure showed features similar to the internal jellyfish-like structure of *M. mobile* (5, 12) (Movie S2, Fig. S1C). We compared the features of particle structures before and after the removal of the cell membrane (Fig. S1D). After removal, the height of the particle relative to the background increased, resulting in a clearer appearance than before removal. The particle pitches were 30.3 ± 4.1 and 31.8 ± 7.3 nm before and after removal, respectively, without a statistically significant difference (*P = *0.277 by Student’s *t* test). The average heights of particles observed before and after removal of the cell membrane were 257 and 18 nm, respectively, from the lowest position of the image. The difference between them was 239 nm, comparable to the height of *M. mobile* cells (Fig. 2A and Fig. S1D). Therefore, the particles detected before and after cell membrane removal were proposed to be the structure beneath the upper cell membrane and the one on the lower cell membrane facing the glass substrate, respectively. This occasional observation is likely related to the character of *M. mobile* surface structure, that is a soft single-layered membrane (5). However, we could not remove the cell membrane intentionally. Then, we focused on analyzing the internal structure beneath the upper cell membrane.

10.1128/mBio.00040-21.4 MOVIE S2 HS-AFM movie showing removal of the cell membrane. The upper membrane of a cell scanned at 3 fps was removed at approximately 20 s. The video was played at 5× speed. The scanning field was 500 by 500 nm^2^ with 150 by 150 pixels. The cell front is directed to the upper right. Download Movie S2, AVI file, 2.7 MB. Copyright © 2021 Kobayashi et al. 2021 Kobayashi et al. https://creativecommons.org/licenses/by/4.0/ This content is distributed under the terms of the Creative Commons Attribution 4.0 International license.

### Behavior of particle structure detected by HS-AFM.

The surface protrusion of *M. mobile* cells was scanned with a scanning rate of 200 or 330 ms per frame with a scan area of 200 by 200 nm^2^. Projected images were processed using a bandpass filter to improve the image contrast, by drift correction, and by averaging three sequential images for better signal/noise ratio (Movie S4). In most cases, the particles were difficult to trace over time because of image discontinuity, even when particle images were clear. This is probably due to the stability of the cell immobilized onto the glass surface and damage to the scanning probe. However, we succeeded in tracing the behaviors of individual particles in some videos and used them for further analyses.

10.1128/mBio.00040-21.6 MOVIE S4 HS-AFM movie showing particle movements. The cell surface was scanned at 5 fps. The scanning field was 200 by 200 nm^2^ with 100 by 100 pixels. The video was played at 2× speed. The cell front is directed to the upper right. Download Movie S4, AVI file, 0.8 MB. Copyright © 2021 Kobayashi et al. 2021 Kobayashi et al. https://creativecommons.org/licenses/by/4.0/ This content is distributed under the terms of the Creative Commons Attribution 4.0 International license.

### Sodium azide suppressed particle movement.

**FIG 3:**
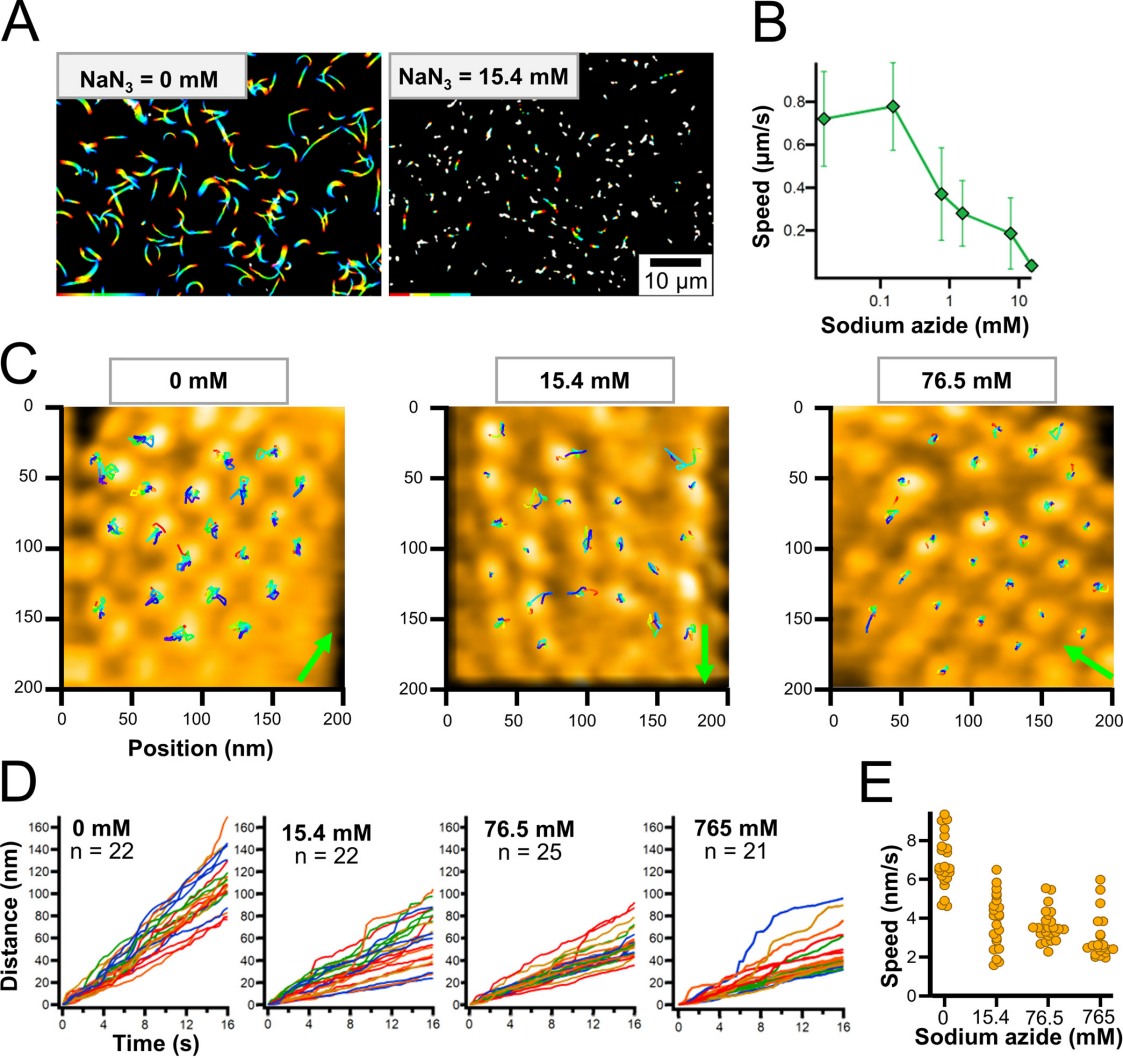
Effects of sodium azide on particle displacements. (A) Rainbow traces of gliding cells for 5 s with and without sodium azide from phase-contrast microscopy. Video frames were overlaid with different colors from red to blue. (B) Gliding speed under various concentrations of sodium azide. Speeds of 2.5 to 20 s were averaged for 140 to 223 cells. (C) HS-AFM images with continuous traces of individual particles for 13.2 s. HS-AFM images were processed by bandpass filter, drift correction, and sequential averaging. Particles were traced every 200 ms for no sodium azide, and 330 ms in the presence of sodium azide, as presented by the color change from red to blue. The cell axis and front are indicated by a green arrow. The surface was scanned left to right for line and lower to upper for imaging. Movies are shown as supplemental data as Movies S4, S5, S6, and S7 for imaging in 0, 15.4, 76.5, 765 mM sodium azide, respectively. (D) Time course of accumulated moving distances of individual particles under various concentrations of sodium azide. (E) Scatter dot plot of particle speed under various concentrations of sodium azide. Speeds were estimated from a linear fitting of accumulated moving distance.

To discuss the behaviors of internal particles, we needed to confirm that the particle movements are caused by ATP hydrolysis on the internal structure. In a previous study, the ATPase activity of the internal structure of *M. mobile* was inhibited by sodium azide (5). The binding activity and gliding speed of “gliding heads,” the gliding machinery isolated from the cell protrusion, were also inhibited by sodium azide (5). In the present study, we examined the effect of sodium azide on the gliding speed of intact *M. mobile* cells. The averaged gliding speed of intact *M. mobile* cells was decreased from 0.77 ± 0.17 to 0.04 ± 0.02 μm/s by the addition of 15.4 mM sodium azide (Fig. 3A and B), suggesting that sodium azide affected the ATPase activity of the internal structure and the force generation for gliding.

10.1128/mBio.00040-21.7 MOVIE S5 HS-AFM movie showing particle movements under 15.4 mM sodium azide. The cell surface was scanned at 3 fps. The scanning field was 200 by 200 nm^2^ with 100 by 100 pixels. The video was played at 3.3× speed. The cell front was directed to the lower portion of the frame. Download Movie S5, AVI file, 0.6 MB. Copyright © 2021 Kobayashi et al. 2021 Kobayashi et al. https://creativecommons.org/licenses/by/4.0/ This content is distributed under the terms of the Creative Commons Attribution 4.0 International license.

10.1128/mBio.00040-21.8 MOVIE S6 HS-AFM movie showing particle movements under 76.5 mM sodium azide. The cell surface was scanned at 3 fps. The scanning field was 200 by 200 nm^2^ with 100 by 100 pixels. The video was played at 3.3× speed. The cell front is directed to the upper left. Download Movie S6, AVI file, 0.7 MB. Copyright © 2021 Kobayashi et al. 2021 Kobayashi et al. https://creativecommons.org/licenses/by/4.0/ This content is distributed under the terms of the Creative Commons Attribution 4.0 International license.

10.1128/mBio.00040-21.9 MOVIE S7 HS-AFM movie showing particle movements under 765 mM sodium azide. The cell surface was scanned at 3 fps. The scanning field was 200 by 200 nm^2^ with 100 by 100 pixels. The video was played at 3.3× speed. The cell front is directed to the upper left. Download Movie S7, AVI file, 0.6 MB. Copyright © 2021 Kobayashi et al. 2021 Kobayashi et al. https://creativecommons.org/licenses/by/4.0/ This content is distributed under the terms of the Creative Commons Attribution 4.0 International license.

We then scanned the cell surfaces by HS-AFM in the presence and absence of sodium azide (Movie S4 to S7). The tracking of the mass center every 200 ms (no azide) or 330 ms (with azide) for 16.2 s showed that most particles were moving independently (Fig. 3C). These movements were significantly reduced by the addition of sodium azide. We calculated the accumulated moving distances and estimated the speeds for the particle movements from a linear fitting of the accumulated moving distance (Fig. 3D and E). At concentrations of 0, 15.4, 76.5, and 765 mM sodium azide, the speeds calculated from accumulated moving distances were 6.9 ± 1.4, 3.9 ± 1.4, 3.6 ± 0.8, and 3.0 ± 1.1 nm/s, respectively, suggesting that the movement of particle structures is linked to ATP hydrolysis. Interestingly, in 15.4 mM sodium azide, the particles can be classified as either active or static, and the different types tend to form an adjacent pair in chains (Fig. 3C).

### Particle displacements traced as an image profile.

**FIG 4:**
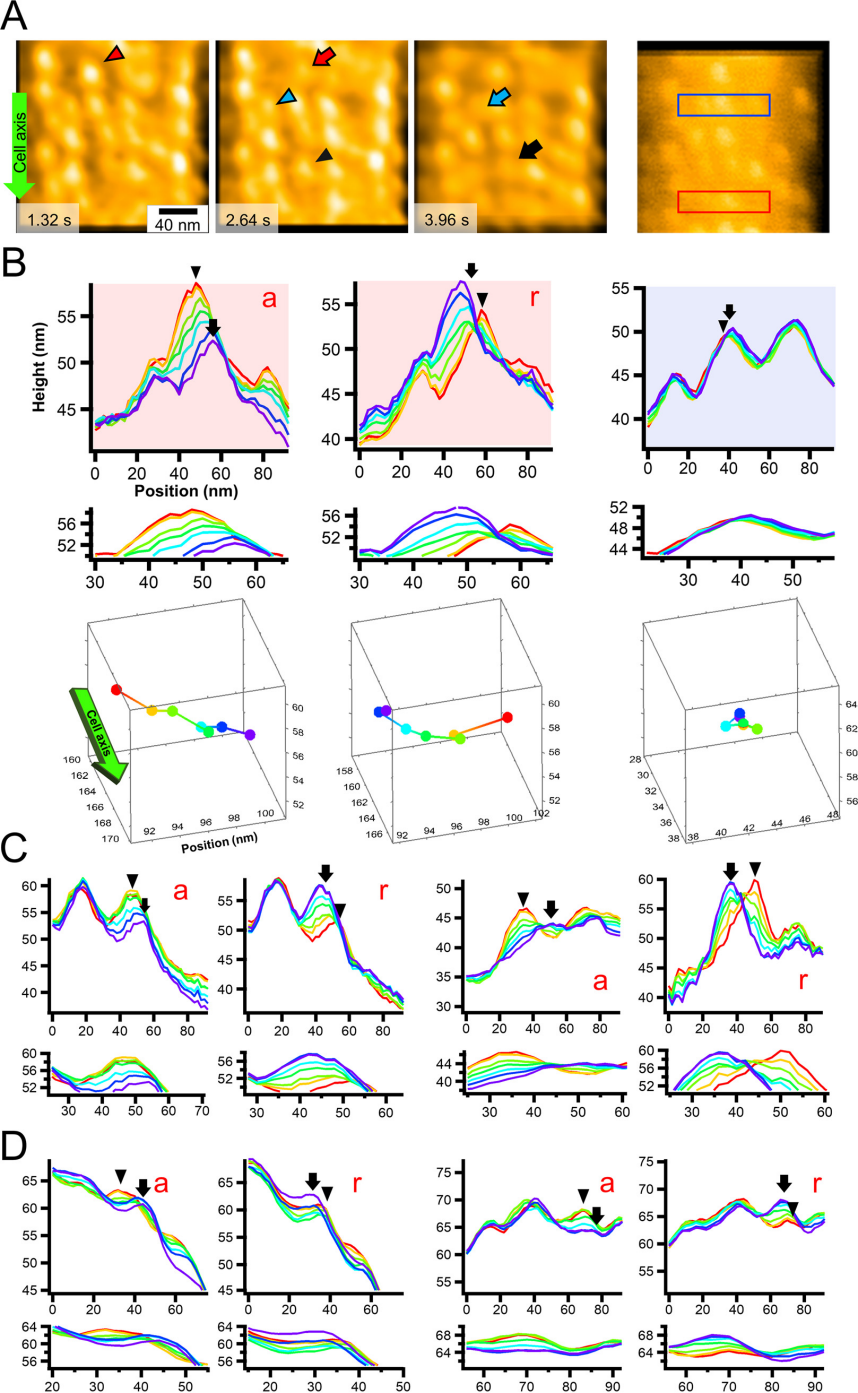
Movements of individual particles. (A) Video frames of particle chains under 15.4 mM sodium azide (Movie S5). The green arrow on the left shows the cell axis and front. The left three panels show consecutive video frames showing remarkable particle movements. The particles with movements are marked before (triangles) and after (arrows) the movements with coordinated colors. Particles moved to the left relative to the gliding direction. The rightmost panel shows a raw image of the video frame showing areas profiled for active (red) and static (blue) particles shown in panel B. (B) Consecutive image profile of representative active and static particles. (Upper six graphs) Image profiles of active (red background) and static (blue background) particles every 330 ms for 1.98 s. (Lowermost graphs) Three-dimensional positions of peaks of particles tracing from red to purple. Y positions are shown only in these graphs in Fig. 4. The green arrow on the left shows the cell axis and front. (C) Consecutive image profiles showing particle movements every 330 ms for 1.98 s in 15.4 mM sodium azide. (D) Consecutive image profiles showing particle movements every 200 ms for 1.2 s without sodium azide (Movie S4). (B to D) Consecutive profiles of each frame from red to purple. Advancing (a) and returning (r) movements are presented. Peak positions of focusing particles are marked by a triangle and an arrow, respectively, for the initial and the end time points. Distances between peaks before and after movement were manually measured for statistical analysis of particle movements. The profile of heights and positions is presented with a common X- Y- scale in the lower panel for each data set.

Not all particles moved in the same direction at the same time (Fig. 3C to E), and this feature was more obvious in 15.4 mM sodium azide (Movie S5, Fig. 4A), indicating that the movements were linked to ATP hydrolysis, not caused by artificial drift in the measurements. The addition of sodium azide may allow easier detection of individual movements by reducing some of the movements. Analysis of 27 particles in a 200 by 200 nm^2^ field in the presence of 15.4 mM sodium azide for 23.1 s showed that 19 particles moved distances longer than 6 nm, distinct from other movements. The frequency of such long movements in the whole field was 1.17 events/s (Fig. 4A). Next, we focused on particle movements. Since the particles appeared to move mainly perpendicular to the particle chain in the cell surface plane, the height profile of a box perpendicular to the particle chain was traced over time (Fig. 4B, upper graphs). Six particles did not move (static particle), while 15 active particles showed remarkable movements, and a returning path for some particles was observed. As shown in the “a” panels of Fig. 4B and C, the movements of the particles showed a tendency moving 9.1 ± 2.5 nm (*n* = 15) in the left direction perpendicular to the chain axis and 2.3 ± 3.0 nm (*n* = 8) on the cytoplasmic side in the Z direction. These behaviors can be traced three-dimensionally as shown for the representative particle movements (Fig. 4B, lowermost graphs). The profile continued to change for approximately five frames of 330 ms. However, the movement was likely completed in a single 330-ms frame, because the image was profiled after averaging three consecutive video images every 330 ms to reduce image noise. Eleven particles showed returning movements in the video, with speeds similar to those of their advancing movements, as shown in the panels marked “r” in Fig. 4B and C. In conclusion, the active particles moved to 9 nm left and 2 nm lower relative to the cell axis in 330 ms and came back to the original position in another 330 ms.

Next, particle movements perpendicular to the cell axis were searched in the absence of sodium azide. Observation of 21 particles for 16.6 s showed that movements longer than 6 nm appeared at a frequency of 2.17 events/s (Movie S4 and Fig. 4D). The distance moved was 8.0 ± 1.9 nm (*n* = 24) in the left direction perpendicular to the axis of the chain alignment within 200 ms and 2.0 ± 1.9 nm (*n* = 18) on the cytoplasmic side in the Z direction (Fig. 4D).

### Particle displacements traced as a positional distribution.

**FIG 5:**
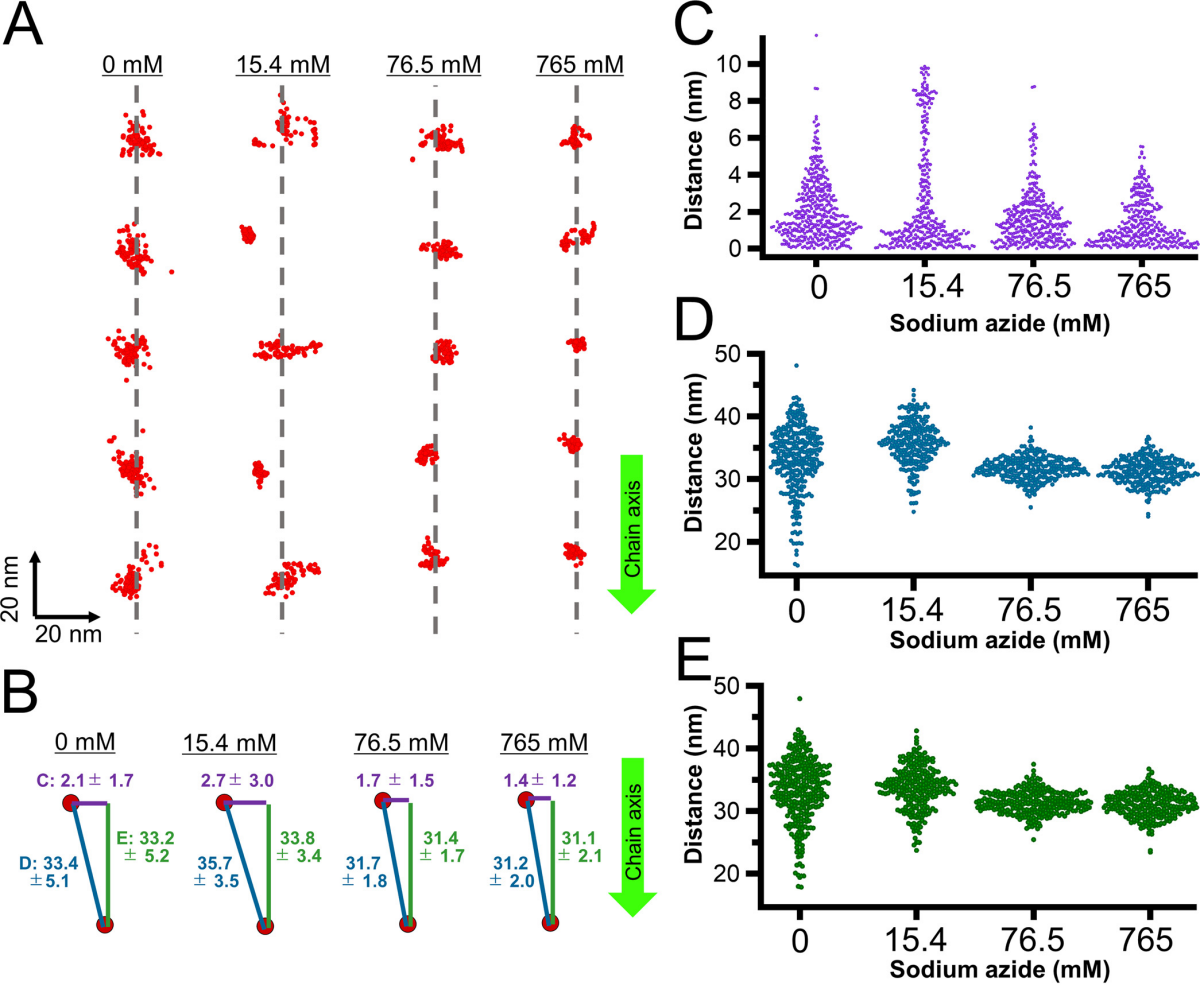
Analyses of particle distribution. (A) Distribution of particles in a chain. The particle positions and the axis of the particle positions are indicated by red dots and gray dashed lines, respectively. The particle positions were detected every 200 and 330 ms, respectively, without and with sodium azide at 82, 66, 70, and 66 points under 0, 15.4, 76.5, and 765 mM sodium azide, respectively. The axis of particle positions was determined by a linear approximation of the average position of each particle. (B) Schematic illustration of three distances with average and standard deviation (SD) values in nm. (C to E) The particle position to the chain axis (C, purple), the distance to the adjacent particle (D, blue), and the distance to the adjacent particle projected to the chain axis (E, green) are shown. Bar lengths are not to scale. Movies S4 to S7 were analyzed. The chain axis is indicated by a green arrow pointing mostly to the cell front in panels A and B.

To study the direction of movements of the particles on the membrane surface statistically, the distributions of the particles as the mass center were analyzed every 200 and 330 ms for observations in the absence and presence of sodium azide, respectively (Fig. 5A and Movies S5 to S7). In this analysis, we determined the particle positions for the *x* and *y* axes (Fig. 5A), while only the *x* and *z* axes were shown in panels C and D of Fig. 4. Instead, we did not trace the particle positions with time in Fig. 5. The faster scan speed for the observation in the absence of sodium azide was applied, as we assumed that the particles moved faster under these conditions. However, this difference in the scanning speed should not affect the conclusion, because no difference was found, even when the analysis was performed using 400-ms intervals for the measurements without sodium azide (Fig. S2). Analysis showed that the distributions were larger in the presence of 15.4 mM and smaller at 76.5 and 765 mM than in the absence of sodium azide (Fig. 5A). Next, we measured the distributions of three distances (Fig. 5B) as follows: the particle position to the chain axis (Fig. 5C), the distance to the adjacent particle (Fig. 5D), and the distance to the adjacent particle projected to the chain axis (Fig. 5E). These results are schematically summarized (Fig. 5B), suggesting that movements perpendicular to the chain axis of the particles (presented as distance “c” in Fig. 5) should be present but not easy to detect in the absence of sodium azide; they were observed more clearly when the frequency of movements was reduced by sodium azide, and they were inhibited under high concentrations of sodium azide.

10.1128/mBio.00040-21.2 FIG S2 Particle distribution without sodium azide analyzed using different time intervals. (A to D) Distribution of particles in a chain (A), the particle position to the chain axis (B), the distance to the adjacent particle (C), and the distance to the adjacent particle projected to the chain axis (D) were analyzed every 200, 400, and 600 ms. Download FIG S2, PDF file, 0.2 MB. Copyright © 2021 Kobayashi et al. 2021 Kobayashi et al. https://creativecommons.org/licenses/by/4.0/ This content is distributed under the terms of the Creative Commons Attribution 4.0 International license.

## DISCUSSION

### Internal structure was traced from the outside surface.

The particle features traced by HS-AFM in this study were consistent with those of the internal structure reported in previous studies (Fig. 2) (5, 12), suggesting that HS-AFM visualized the internal structure. The large surface proteins Gli521, Gli349, and Gli123 exist on the cell surface of *M. mobile* as components of the gliding machinery (7, 14, 17–20, 22, 24, 49). A group of surface proteins, Mvsps, which are responsible for antigenic variations, also exist on the cell surface (3, 50). These surface proteins may interfere with probing the internal structure from the surface. However, the chain structures observed by HS-AFM did not show obvious differences before and after protease treatment of the cells (Fig. S1B). Furthermore, similar structures were observed before and after mechanical removal of the cell membrane (Fig. S1C and D). These results showed that the particles traced by HS-AFM were not on the surface structure, but were inside the cell. The surface structure, composed of mainly large filamentous proteins, may be too thin and/or mobile to be detected by the current scanning performance of HS-AFM on the cell membrane (16–18, 22). The lack of a peptidoglycan layer should be advantageous for visualizing the inside structure, due to the lack of stiffness (44, 45, 51). Moreover, the internal structure should be sufficiently stiff and positioned beneath the cell membrane, reminiscent of cortical actin in animal cells (42).

### Effects of sodium azide.

Sodium azide inhibits many ATPases by blocking ADP release (52). In *M. mobile* gliding, the reagent inhibited cell gliding (Fig. 3A and B) and the isolated gliding machinery (5). Particle behaviors became more visible in the presence of 15.4 mM sodium azide. Under this condition, cell gliding was reduced to 20 times slower than the original, suggesting that ATP hydrolysis occurred 20 times less frequently. If the particles move in a rapid and independent manner, it may be difficult to trace the movements of individual particles. However, if the reaction was partially inhibited by 15.4 mM sodium azide, most particles may be in their home position, while some particles move to another position. In this case, the movements could be traced easily. This assumption is supported by the observation that the particle distances between neighboring particles are 1.7 to 2.5 nm shorter under high concentrations of sodium azide than those without the reagent (Fig. 5D). A previous study based on electron microscopy showed that the particle distances in the ADP and unbound forms were approximately 2 nm shorter than those in the AMPPNP, ADP-V_i_, and ADP-AlF_x_ states (5). As sodium azide is thought to inhibit the release of ADP (52), the changes in particle distance observed in the present study are consistent with the results of electron microscopy (Fig. 5B) (5).

### Particle behavior in the gliding mechanism.

**FIG 6:**
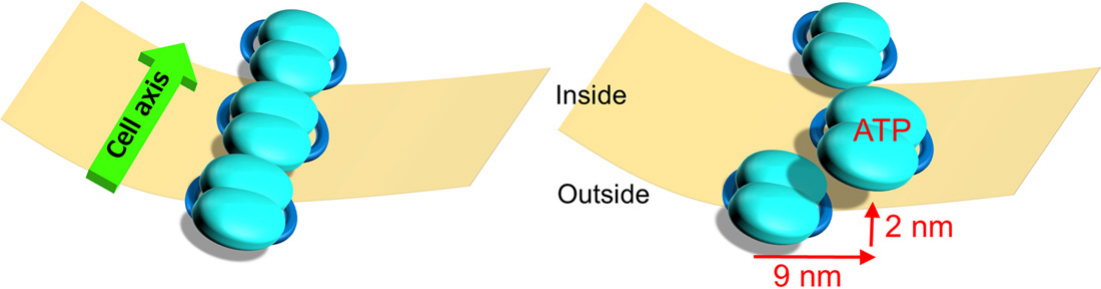
Schematic illustration of particle movement in *M. mobile* visualized by HS-AFM. The internal chain of the gliding machinery and cell membrane are indicated by blue objects and a beige plate, respectively. Here, we focus on the particle chain lining the lower side of cell membrane, while we scanned mostly the particle chain beneath the upper side of the cell membrane in this study. The left and right panels show the particles before and after the advancing movement, respectively. The central particle moves as an ATP- or ADP/P_i_-bound form to the right and inner sides for a distance of 9 and 2 nm, respectively.

The particles moved approximately 9 nm to the right of the gliding direction and 2 nm to the cytoplasmic side within 330 ms (Fig. 6). This movement may be coupled with the transition from ADP or unbound form to ATP or the ADP/P_i_ form (5). Considering the fact that the particles are structurally linked to the surface structures of the gliding machinery (5), the movements observed in the present study are likely involved in the gliding mechanism.

There are two possibilities for the relation between the particle movements and gliding motility. As *M. mobile* gliding is caused probably by repetitive leg strokes (21, 29, 32), the particle movements may be coupled to the stroke. Previous studies have reported that the step size of *M. mobile* is approximately 70 nm under no load and adjustable to various loads (21, 29, 32, 53). The moving distances of particles, approximately 10 nm is much shorter than the step size. However, this difference can be explained because the surface structure contains two large proteins with dimensions comparable to the step size; that is, the Gli349 “leg” that catches the scaffold and the Gli521 “crank” that transmits force for gliding are 100 and 120 nm long, respectively (Fig. 1A) (16, 17, 22). Therefore, the movements occurring in the internal structure can be amplified through the huge protein molecules on the surface or through an unknown structure that connects the internal and surface structures (Fig. 1A and 2G). This assumption can explain the previous observation that the single leg exerts a force of 1.5 pN, a few times smaller than motor proteins (21), assuming elastic components are equipped in the large surface complex. Another possibility is that the movements observed here are caused as a “reaction” of cooperating many particles in a large complex, besides a direct transmission linking the particle and the surface structures. In this scenario, the movements of the transmission were too small to be detected by HS-AFM, for example rotation of rod proteins.

In a previous study, *M. mobile* gliding showed a leftward directional change of about 8.5° with 1-μm cell progress (31). This gliding property may be related to the observation that the particle movements are pointed to the right relative to the gliding direction (Fig. 6). Otherwise, the tilting of the chain axis about 4.6° from the cell axis may cause a directional change in gliding (Fig. 2D).

To elucidate the mechanism of *M. mobile* gliding, we need to further visualize the behaviors and structures of the machinery in detail, including those of both internal and surface structures. The combination of electron microscopy and HS-AFM may provide better insights in the near future.

## MATERIALS AND METHODS

### Cell preparation.

A mutant strain (*gli521*[P476R]) of *M. mobile* 163K (ATCC 43663) activated for binding (21, 23, 54) was grown in Aluotto medium at 25 to 28°C, as previously described (8, 49). Cultured cells were collected by centrifugation at 12,000 × *g* for 4 min at 25 to 28°C and suspended in phosphate-buffered saline with glucose (PBS/G) consisting of 75 mM sodium phosphate (pH 7.3), 68 mM NaCl, and 10 mM glucose (21, 26, 30, 31). This process was repeated twice, and finally the cells were resuspended in PBS/G to a 20-fold density of the original culture.

### Gliding analyses.

A tunnel chamber assembled as previously described (3-mm interior width, 22-mm length, 40-μm wall thickness) was treated with Aluotto medium for 15 min at 25 to 28°C (21, 30), and then the medium was replaced by PBS/G. The cell suspension was inserted into the tunnel chamber with video recording. PBS/G was replaced with PBS/G containing 0.2 mg/ml proteinase K (Qiagen N. V., Hilden, Germany) or various concentrations of sodium azide, as necessary.

### Cell immobilization on the glass surface.

A glass slide was treated with saturated KOH-ethanol solution for 15 min and washed 10 times with water. For analyses with an imaging rate of 1,000 and 330 ms per frame, the glass was treated with 0.1% poly-l-lysine for 5 min. After the solution was removed, the glass was washed with water and dried. Then, the glass was treated with 0.1% glutaraldehyde for 5 min, washed with water, and covered with PBS/G. For analyses with an imaging rate of 200 ms per frame, the glass was treated with sandpaper, saturated with KOH-ethanol solution for 15 min, washed 10 times with water, and then dried. The washed glass was treated with 1,000-fold diluted 3-aminopropyldiethoxymethylsilane for 5 min at 25 to 28°C, washed, and treated with glutaraldehyde as described above. Finally, the cell suspension was placed onto the glass substrate and left for 10 min at 25 to 28°C.

### Microscopy.

To examine the immobilizing conditions using phase-contrast microscopy, the glass slide was assembled into a tunnel chamber (19). The cell suspension was loaded into the tunnel, kept for 10 min at 25 to 28°C, washed with PBS/G, and observed by phase-contrast microscopy IX71 (Olympus, Tokyo, Japan) (21, 27, 31). To analyze the immobilizing conditions, quick-freeze deep-etch electron microscopy, fixation, and washing were performed on the coverslip. When the cells were frozen without immobilization, we followed the procedure for the electron microscopy method described previously (44, 45). Briefly, the cells on the glass were pressed against a copper block cooled with liquid helium and frozen. Then, the frozen sample was fractured and etched to expose it. Subsequently, the exposed surface was shadowed with platinum to create a replica membrane, which was observed under a JEM-1010 transmission electron microscope (JEOL, Tokyo, Japan) at 80 kV, equipped with a FastScan-F214 (T) charge-coupled device (CCD) camera (TVIPS, Gauting, Germany).

### Observation by HS-AFM.

Imaging was performed with a laboratory-built HS-AFM in tapping mode (55, 56). Small cantilevers (BLAC10DS-A2; Olympus) with a resonant frequency of ∼0.5 MHz in water, a quality factor (*Q*_c_) of ∼1.5 in water, and a spring constant (*k*_c_) of ∼0.08 N/m were used. The cantilever’s free oscillation amplitude (*A*_0_) and set-point amplitude (*A*_sp_) were set at ∼2.5 nm and ∼0.8 × *A*_0_, respectively. Under these conditions, the average tapping force <*F*> can be approximated as ∼40 pN using the following equation:

$$F=\frac{k_{c}}{2Q_{c}}\sqrt{A_{0}^{2}-A_{sp}^{2}}$$

For searching cells, the sample was scanned at an imaging rate of 1,000 ms per frame in an area of 3,000 by 3,000 nm^2^ with 150 by 150 pixels. To observe the particle structure, the cell surface was scanned with an imaging rate of 330 or 200 ms per frame in an area of 200 by 200 nm^2^ with 100 by 100 pixels.

### Video analyses.

To trace particles in the XY plane, videos were processed by three methods (Movies S3 to S7). (i) The image contrast was improved by a bandpass filter. (ii) Image drifts were corrected by a plugin, “align slices in stack” (57), equipped with ImageJ. (iii) Image noises were removed by averaging three consecutive slices. Then, each particle image was cropped, binarized, and traced for the mass center. Here, the threshold for binarization was determined independently for each particle of interest. The cell axes in Fig. 2 were determined by fitting a cell image as an ellipse. All analyses were performed with ImageJ version 1.52A. Image averaging of particles was performed using EMAN, version 2.3.

10.1128/mBio.00040-21.5 MOVIE S3 Effects of image processing on HS-AFM movies of the cell surface. The original movie (upper left) was processed using a bandpass filter (upper right), bandpass filter + drift correction (lower left), and bandpass filter + drift correction + sequential averaging (lower right). The video was played at 3.3× speed. The cell front was directed to the lower portion of the frame. Download Movie S3, AVI file, 2.7 MB. Copyright © 2021 Kobayashi et al. 2021 Kobayashi et al. https://creativecommons.org/licenses/by/4.0/ This content is distributed under the terms of the Creative Commons Attribution 4.0 International license.
