# Supplementary figures and images for: Movements of Mycoplasma mobile Gliding Machinery Detected by High-Speed Atomic Force Microscopy
Source: mBio. 2021 May 28;12(3):e00040-21. doi: 10.1128/mBio.00040-21 (PMC8262943; doi:10.1128/mBio.00040-21)

A

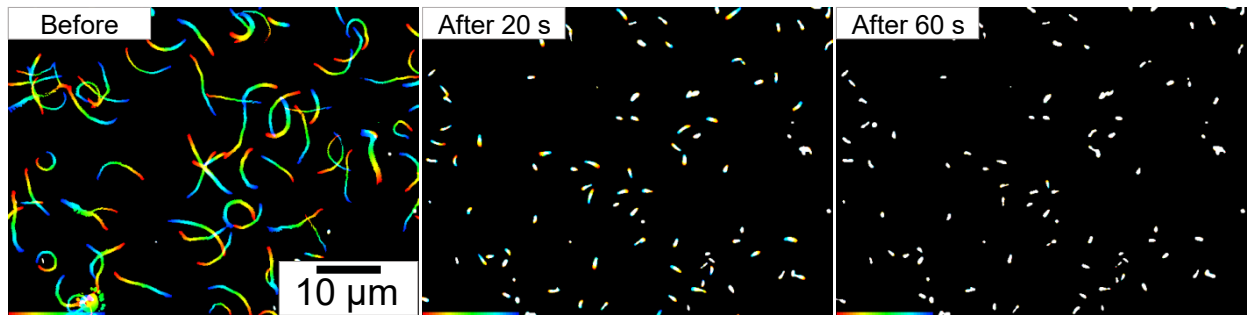

B

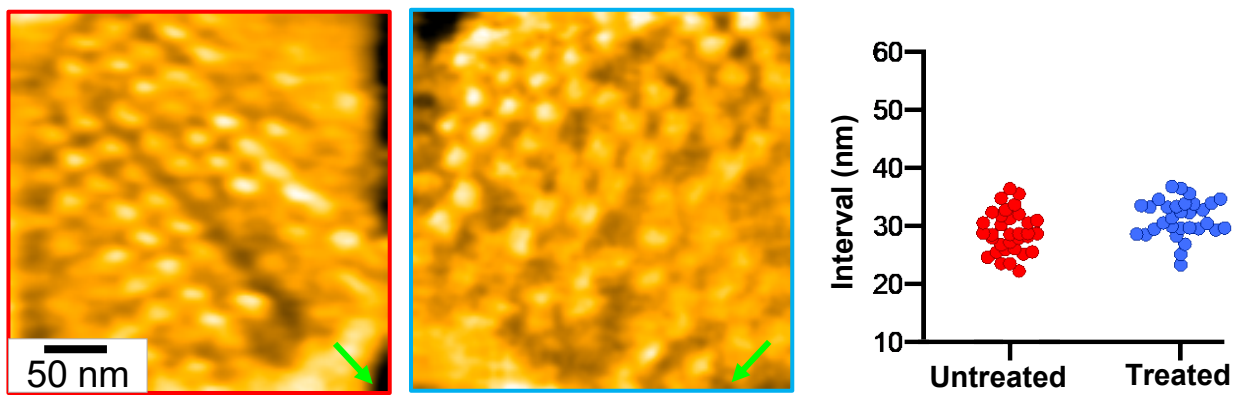

C

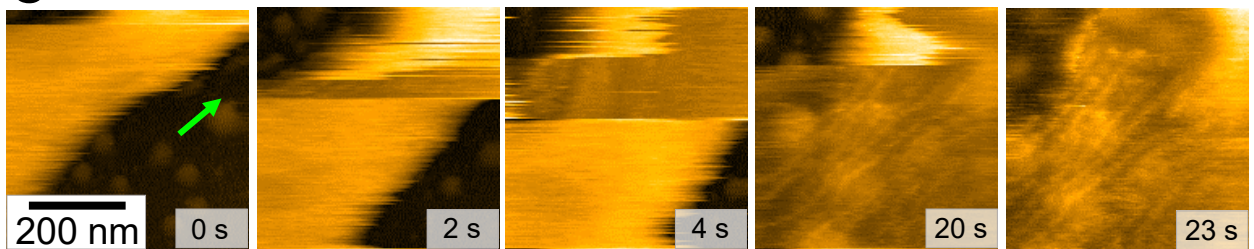

D

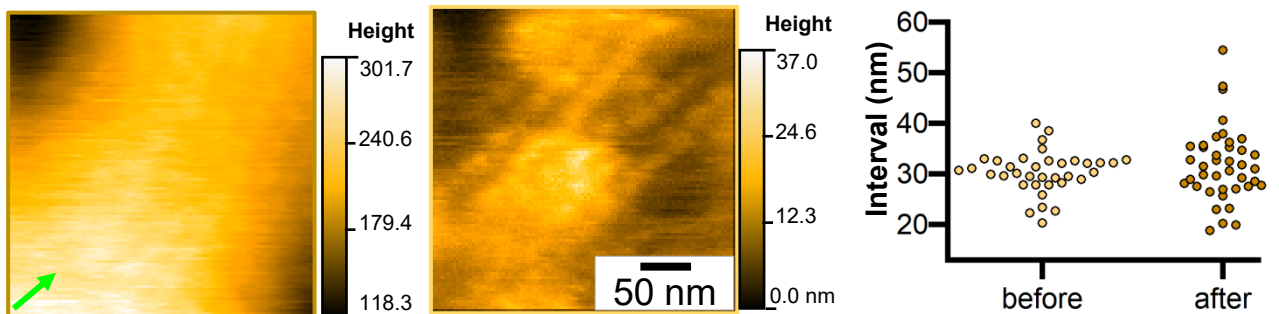

Supplement: FIG S1 [file mbio.00040-21-sf001.pdf]
