# Supplementary material for: Movements of Mycoplasma mobile Gliding Machinery Detected by High-Speed Atomic Force Microscopy
Source: mBio. 2021 May 28;12(3):e00040-21. doi: 10.1128/mBio.00040-21 (PMC8262943; doi:10.1128/mBio.00040-21)
Supplement: FIG S2 [file mbio.00040-21-sf002.pdf]

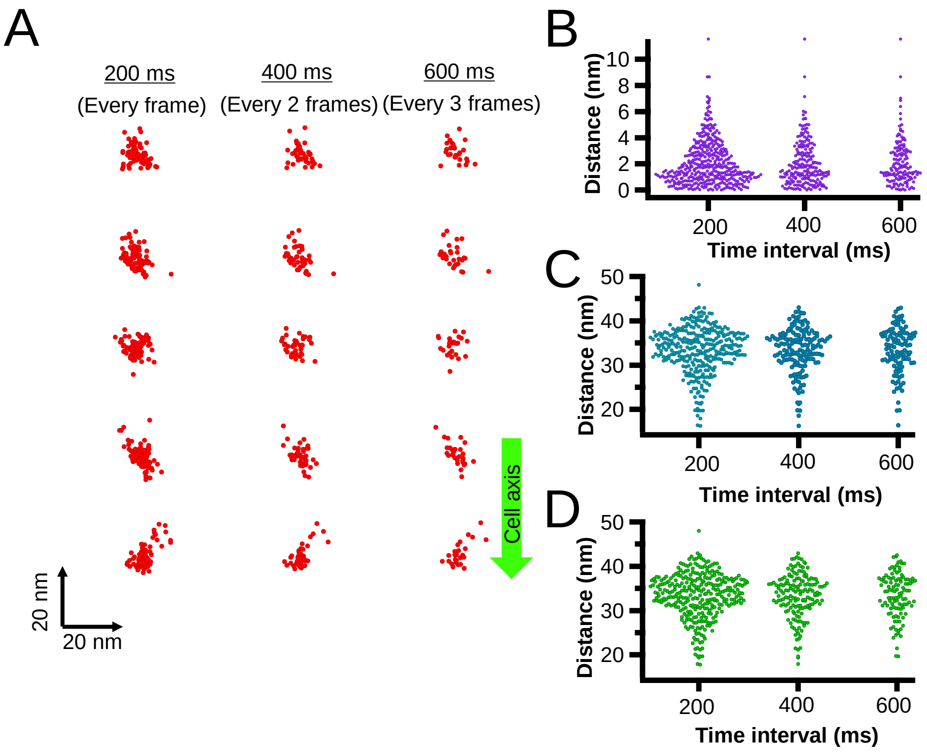

FIG S2 Particle distribution without sodium azide analyzed using different time intervals. Distribution of particles in a chain (A), the particle position to the chain axis (B), the distance to the adjacent particle (C), and the distance to the adjacent particle projected to the chain axis (D) were analyzed every 200, 400, and 600 ms.
